# AMRnet: a data visualization platform to interactively explore pathogen variants and antimicrobial resistance

**DOI:** 10.1093/nar/gkaf1101

**Published:** 2025-11-06

**Authors:** Louise T Cerdeira, Zoe A Dyson, Vandana Sharma, Mary Maranga, Ebenezer Foster-Nyarko, Megan E Carey, Kathryn E Holt

**Affiliations:** Department of Infection Biology, Faculty of Infectious and Tropical Diseases, London School of Hygiene and Tropical Medicine, London WC1E 7HT, United Kingdom; Department of Infection Biology, Faculty of Infectious and Tropical Diseases, London School of Hygiene and Tropical Medicine, London WC1E 7HT, United Kingdom; Wellcome Sanger Institute, Wellcome Genome Campus, Hinxton CB10 1SA, United Kingdom; Department of Infection Biology, Faculty of Infectious and Tropical Diseases, London School of Hygiene and Tropical Medicine, London WC1E 7HT, United Kingdom; Department of Infection Biology, Faculty of Infectious and Tropical Diseases, London School of Hygiene and Tropical Medicine, London WC1E 7HT, United Kingdom; Department of Infection Biology, Faculty of Infectious and Tropical Diseases, London School of Hygiene and Tropical Medicine, London WC1E 7HT, United Kingdom; Department of Infection Biology, Faculty of Infectious and Tropical Diseases, London School of Hygiene and Tropical Medicine, London WC1E 7HT, United Kingdom; Department of Infection Biology, Faculty of Infectious and Tropical Diseases, London School of Hygiene and Tropical Medicine, London WC1E 7HT, United Kingdom; Department of Infectious Diseases, School of Translational Medicine, Monash University, Melbourne, Victoria 3004, Australia

## Abstract

Antimicrobial resistance (AMR) poses a substantial threat to global public health. Whole genome sequencing is increasingly used as a core method for pathogen characterization to support AMR surveillance. As a result, a vast amount of bacterial sequence data are available in public archives, yet the AMR-related information they encode is not readily accessible to those without bioinformatics expertise and is essentially invisible to policy makers. The AMRnet platform aims to make publicly available genome-derived AMR data accessible to a diverse user base. The underlying data are drawn from public genomic databases and used to calculate pooled estimates of national annual prevalence that can be visualized interactively, and broken down and explored in terms of underlying pathogen variants, resistance mechanisms, and geographic and temporal distributions. Users can download dynamically generated reports, summary and line-list data from the web-based dashboard (https://www.amrnet.org), and query the database via application programming interface. For selected organisms, data are curated for purpose of sampling, to reduce the public data bias towards sequencing of resistant or severe infections. By improving the accessibility and utility of publicly archived data, AMRnet aims to encourage wider sequencing initiatives and collaborative data-sharing efforts while providing crucial data insights for researchers and policy makers.

## Graphical Abstract

**Graphical Abstract:**
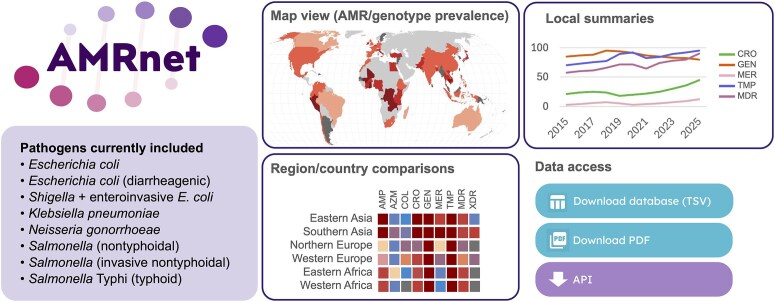

## Introduction

The World Health Organization (WHO) recognizes antimicrobial resistance (AMR) as a leading global health priority, contributing to an estimated 4.95 million deaths in 2019 [1]. It is a complex problem with the potential to compromise the overall safety of healthcare delivery, from surgery to chemotherapy. AMR surveillance, informing on the prevalence of resistance to specific antimicrobials among infections caused by specific pathogens (‘bug–drug’ combinations), is an essential tool to inform AMR policy at national and global levels, as well as to guide infection prevention and control efforts [2]. AMR surveillance data are typically summarized as national annual prevalence estimates for phenotypically determined resistance, for specific bug–drug combinations. Policy makers use the data to inform on a range of clinical and public health issues, including recommendations for the use of drugs for empirical treatment or prevention of infection, policies affecting the availability of requisite drugs, and making the case for national immunization programs and other preventative measures.

Whole genome sequencing is increasingly used as a core method for bacterial pathogen characterization in research and public health settings and has several features that make it attractive for AMR surveillance [3]. Genome-derived AMR data can be richer in information content than phenotype data, providing signals of spatial and temporal dynamics of AMR emergence and spread [4] that can be used to inform policy, complementing AMR prevalence data, whether it be derived from phenotyping or genomic predictions or both [5]. As such, there are many tools to support investigative analysis of genomics data to address AMR at a local level [6], but very few resources to support global comparisons or to make genomics-derived AMR data and insights accessible to a high-level audience of policy makers.

It is a common practice for research and public health laboratories to deposit raw pathogen sequence data in public databases belonging to the International Nucleotide Sequence Database Collaboration (INSDC) [7]. However, specialist bioinformatics expertise and computational infrastructure are needed to make use of these data. Public health focused databases such as PubMLST [8], Enterobase [9], and Pathogenwatch [10–12] provide an additional layer of information, taking raw genome data from INSDC and/or user uploads of specific pathogens of interest; extracting typing information from the genomes, including multi-locus sequence typing (MLST), serotyping, pathotyping, and AMR genotyping; and making that data accessible through interactive websites viewable with standard browsers and data downloads. However, these tools are mostly used by experts in microbiology or bioinformatics and are not geared towards public health epidemiologists or policy makers.

We recently developed a solution for *Salmonella enterica* serovar Typhi (*S*. Typhi, the agent of typhoid fever) in the form of the TyphiNET dashboard [13], which provides interactive visualizations of genome-derived AMR data stratified by bacterial genotype/lineage, using data contributed and curated by the Global Typhoid Genomics Consortium [14], and analysed using the Pathogenwatch platform [10]. AMRnet builds on this approach to provide interactive dashboards for several WHO Priority pathogens [15], drawing genome-derived data from Enterobase and Pathogenwatch, and adding new features including regional and national comparisons, making a wide array of data visualizations and summary data for important AMR pathogens accessible to a diverse audience.

### Architecture

**Figure 1.**
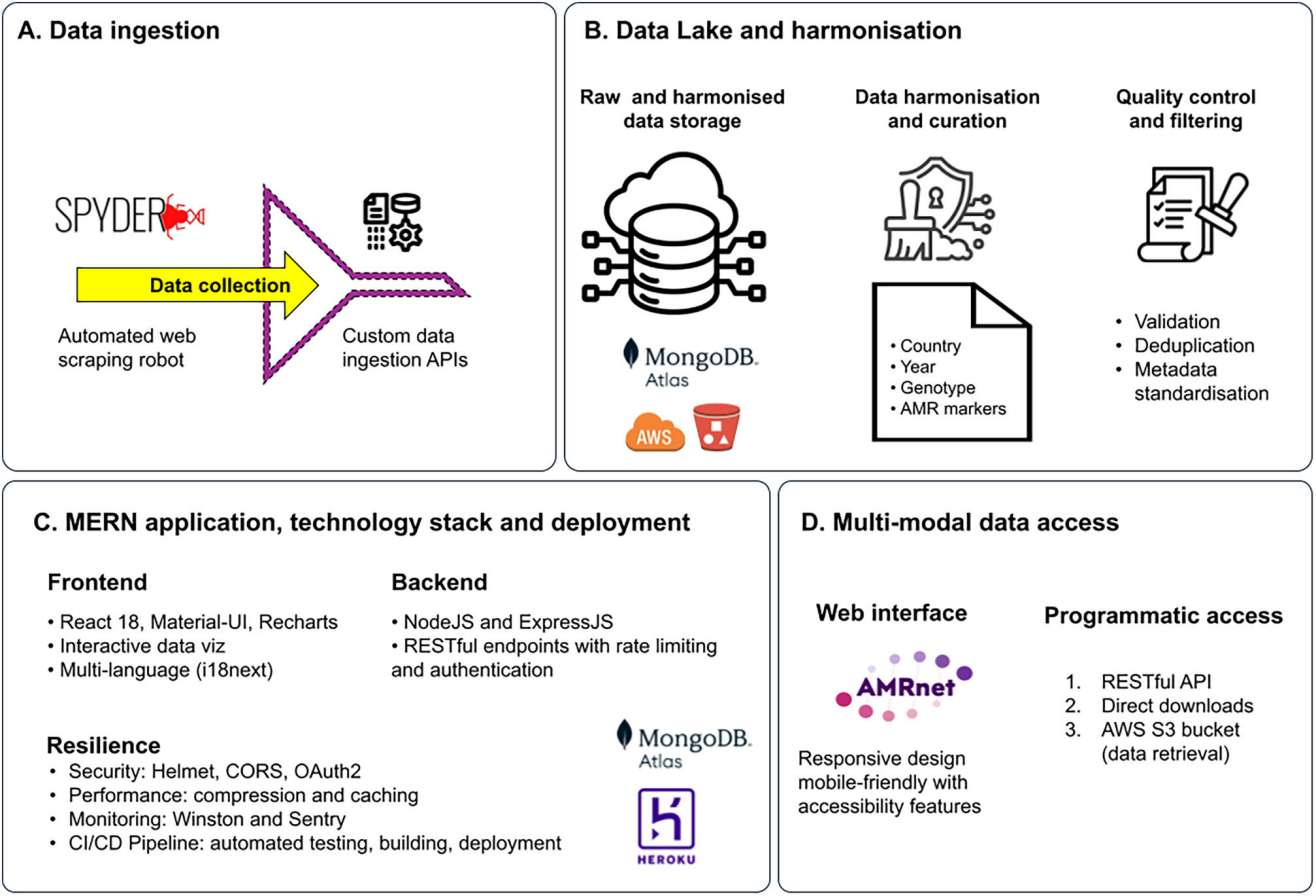
Architecture. The system is built upon a modern and scalable MERN (MongoDB, Express.js, React, Node.js) stack, featuring a four-layer architecture designed to handle large-scale genomic surveillance data while being optimized for performance and user experience. Details of the four layers are illustrated: (**A**) data ingestion, (**B**) data storage and harmonization, (**C**) application and technology stack layer, and (**D**) data access.

The AMRnet web application, available at https://www.amrnet.org, is built using a modern, scalable MERN stack architecture designed to handle large-scale genomic surveillance data (Fig. 1). AMRnet employs a four-layer architecture optimized for performance, scalability, and user experience. The first layer provides automated ingestion of relevant data from public genomic databases (currently Pathogenwatch and Enterobase, see details below). Custom Python scripts, alongside the SPYDER automation tool (https://doi.org/10.5281/zenodo.4619573), are used to ingest data and populate the AMRnet Data Lake. The second layer provides data storage, harmonization, and quality control, using a Software as a Service infrastructure to store both raw and processed data. Data cleaning and harmonization is performed to standardize fields (e.g. country names and date formats) and rectify invalid entries or formatting artefacts. Following this, a quality control process flags samples for inclusion or exclusion. Only samples that pass quality control are integrated into the final version-controlled organism-specific MongoDB collections used by the AMRnet dashboard (see the ‘Database content’ section below). Backup storage and direct data access buckets are provided via Amazon Web Services (AWS) S3 storage. The application layer is constructed as a MERN stack with optimized application programming interface (API) and interactive React frontend, and Node.js and Express.js in the backend. It utilizes robust operational architecture including security protocols, caching mechanisms, system monitoring tools, and a continuous integration/continuous deployment pipeline for automated testing and deployment from the GitHub repository. For deployment, Heroku is used for autoscaling with Fixie proxy for secure database connections. The user access layer provides multimodal access via web interface (interactive dashboard), direct downloads via dashboard buttons, and a RESTful API and AWS S3 bucket access for data retrieval. The web interface is designed for maximum accessibility, featuring responsive layouts for various devices, colour-blind-friendly palettes, and content translated into four languages (using the i18next internationalization framework). The interface allows users to interactively explore and visualize the genomic data (see the ‘Visualizations’ section below).

For each organism, the database contains required input data fields encoding source information (year and country), genotype information [e.g. multi-locus sequence type (ST), lineage, etc. depending on the organism], and AMR marker information (binary indicator fields indicating the presence of each AMR marker). AMR predictions, encoded as binary indicators for each individual drug or class, are also required fields; these may be sourced directly from input data fields (*Neisseria gonorrhoeae*) or calculated from the AMR marker fields using internal logic (all other organisms, see the ‘Database content’ section). For some organisms, additional categorical variables are also calculated internally from the AMR prediction variables, based on established organism-specific definitions [e.g. binary indicators for multidrug resistant (MDR), extensively drug resistant (XDR), or pan-susceptible status]. Currently, MDR and XDR are defined for *S*. Typhi (based on [16]), and *N. gonorrhoeae* [based on European Centre for Disease Prevention and Control (ECDC) definitions [17]]. Finally, additional fields from the input databases may be included for the purpose of filtering (e.g. purpose of sampling and travel versus local origin for *S*. Typhi, and pathotype for *Escherichia coli*; see Table 1). Summary values (numerator, denominator, percentage) are calculated on the fly, on database load and in response to interactive selections made by the user.

**Table 1.** Summary of AMRnet database contents

	Genomes included (/raw)	No. genotypes	No. countries with *N* > 20(with *N* > 100)	Time span(*N* > 100 per year)	Filters
*Escherichia coli*	227 040(/361 803)	11 805 STs (7-locus MLST)	112 (75)	1944–2025(2002–2025)	–
*Escherichia coli* diarrheagenic	68 115(/93 354)	2364 STs (7-locus MLST)	47 [29]	1944–2025(2002–2025)	Pathotype
*Shigella* + EIEC	39, 723(/49 094)	402 lineages (HC400/ST)^a^	43 [21]	1913–2025(2006–2025)	Species/pathotype
*Klebsiella pneumoniae*	41 995 (/48 158)	2, 279 STs (7-locus MLST)27, 752 cgSTs (cgMLST)1231 sublineages (cgMLST)	81 (50)	1935–2023(2003–2023)	ESBL + Carbapenemase +
*Neisseria gonorrhoeae*	19 392(/29 365)	535 STs (7-locus MLST)2089 STs (NG-MAST)	46 [33]	1905–2021(2004–2021)	–
Non-typhoidal *Salmonella*	359 050(/673 149)	1600 lineages (cgMLST)7182 STs (7-locus MLST)	114 (76)	1905–2024(2001–2024)	–
Invasive Non-typhoidal *Salmonella*	13 808(/321 889)	7 lineages (cgMLST)^b^	34 [12]	1905–2024(2001–2024)	Serotype
*Salmonella* Typhi	11 836 (/14 100)	81 GenoTyphi genotypes	30 [18]	1958–2021(2007–2020)	Travel/local onset

a Lineages for *Shigella* are defined using hierarchical cluster (HC) 400 based on core genome multi-locus sequence typing (cgMLST), and for enteroinvasive *E. coli* (EIEC) using 7-locus MLST.

b Lineages defined from HC 150 based on cgMLST, labelled using names proposed in [37] (invasive Typhimurium) and [38] (invasive Enteritidis).

### Database content

**Figure 2.**
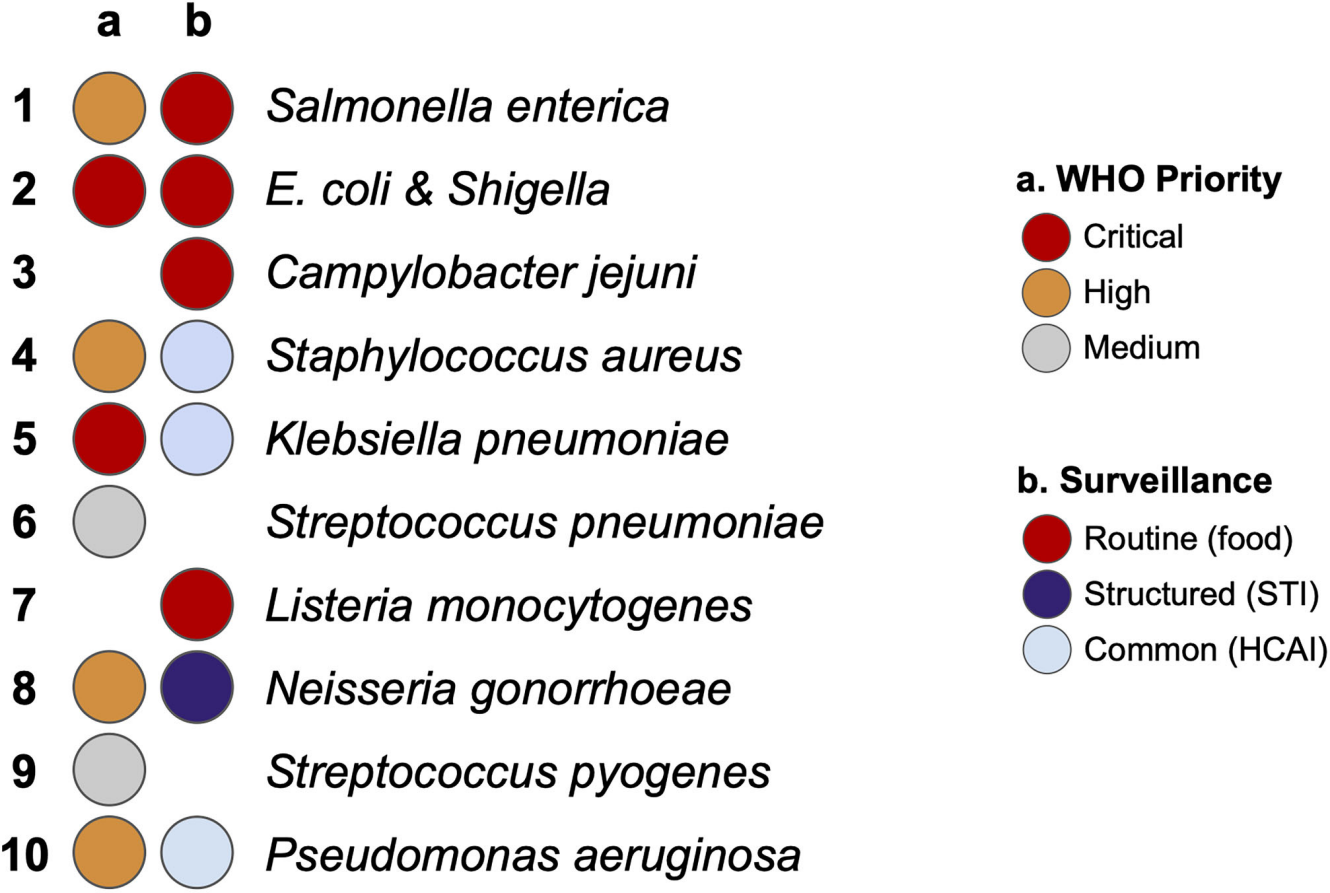
Prioritizing pathogens for inclusion in AMRnet. Top 10 bacterial pathogen species by number of public genome sequences (based on NCBI Pathogen Detection, July 2025 [18]). Coloured circles indicate the WHO Priority level and the status of routine surveillance. Genomic surveillance for *Streptococcus pneumoniae* is increasing, but focused on vaccine serotype rather than AMR.

AMRnet currently includes four bacterial species, selected based on (i) categorization as ‘critical’ or ‘high’ priority in the 2024 WHO Bacterial Priority Pathogens List [15]; (ii) availability of public genome sequence data from diverse geographies; (iii) established use of sequencing for public health surveillance; (iv) availability of genomic frameworks and analysis tools to identify AMR and lineages; and (v) availability of platforms for genome data aggregation, lineage, and AMR typing. Figure 2 shows the top 10 bacterial pathogen species by volume of public sequence data [18], annotated with WHO priority level and current status of sequence-based surveillance. Three of these pathogen species were classified as priority high or critical by WHO and also supported by routine or formally structured genomic surveillance; these include *S. enterica* (both Typhi and non-typhoidal), *E. coli/Shigella*, and *N. gonorrhoeae* [19–21]. All are well served by genomic frameworks and data platforms and were therefore selected for inclusion in the first release of AMRnet. Three common agents of healthcare associated infections (HCAI) were also amongst the top 10 sequenced pathogens: *K. pneumoniae, Staphylococcus aureus*, and *Pseudomonas aeruginosa*. While both *K. pneumoniae* and *S. aureus* are well served by genomic frameworks and data platforms [11, 22, 23], and are common targets for HCAI genomic surveillance in hospital settings [24], we prioritized *K. pneumoniae* for inclusion in AMRnet due to its categorization as a critical priority by WHO and its status as the fastest-growing cause of drug-resistant HCAI deaths [25].

AMRnet databases can, in principle, be populated from any source/s that can provide the required fields (year, country, genotype, AMR markers). Currently, the databases are populated with data imported from either Pathogenwatch (*S*. Typhi, *K. pneumoniae*, and *N. gonorrhoeae*) or Enterobase (*E. coli/Shigella*, and non-typhoidal *S. enterica*). For *E. coli*, AMRnet provides three dashboards, one covering all *E. coli* and excluding *Shigella* spp, and two covering specific sets of pathotypes associated with different types of infections: (i) *Shigella* and EIEC, which invade the human gut epithelium to cause dysentery or shigellosis [26] and (ii) diarrheagenic *E. coli*, comprising the recognized intestinal disease pathotypes EIEC, enterotoxigenic *E. coli*, enterohemorrhagic *E. coli*, shiga toxin-producing *E. coli*, and enteropathogenic *E. coli* [27]. Individual genomes are directed to one or more dashboards based on the genome-derived pathotype classification of each sample, provided by Enterobase. For non-typhoidal *S. enterica*, AMRnet provides two dashboards: one for all non-typhoidal *S. enterica* subsp. *enterica* [based on hierarchical clustering (HierCC) level 2850, HC2850 = 2 [28], and excluding all genomes with predicted serotype Typhi, or Paratyphi A/B/C] and one for invasive lineages of non-typhoidal *S. enterica* serovars Typhimurium and Enteritidis, which together account for >90% of invasive non-typhoidal *Salmonella* (iNTS) globally [29]. The *Salmonella* Typhi (*S. enterica* serovar Typhi) dashboard does not utilize Enterobase but is instead populated from a set of Typhi genome collections in Pathogenwatch that are curated by the Global Typhoid Genomics Consortium as previously described [10, 13, 14]. The *N. gonorrhoeae* dashboard is populated from a set of genome collections in Pathogenwatch corresponding to structured surveillance programs, while the *K. pneumoniae* dashboard is populated from all public *K. pneumoniae* genomes in Pathogenwatch (which currently represents assemblies of all whole genome sequence read sets public in NCBI in March 2023).

The total number of genomes currently in each AMRnet database and corresponding dashboard, the geo-temporal span of included genomes, and the additional filter variables that are available in each dashboard, are summarized in Table 1. The genotype variables available for each dashboard are also indicated; these vary by organism according to current standards for reporting phylogenetic lineages for each pathogen. For *S*. Typhi, genotypes are defined using the GenoTyphi scheme [30, 31] as implemented in Pathogenwatch [10]. For all other organisms, STs based on MLST are available, based on 7-locus or cgMLST schemes [28, 32–34]. For *K. pneumoniae*, sublineages based on cgMLST are also included [35]. For *Shigella*, lineages are labelled with their HierCC level 400 (HC400) types. For *N. gonorrhoeae*, NG-MAST types are also included [36]. For iNTS, lineages defined from HC150 types are included, labelled as proposed in [37] (invasive Typhimurium) and [38] (invasive Enteritidis).

Resistance predictions in AMRnet (binary indicators of resistance to individual drugs or classes) are calculated based on the presence of known AMR markers (except for *N. gonorrhoeae*, where drug-level predictions are imported directly from Pathogenwatch, where they are calculated based on previously described logic [12]). For *S*. Typhi, resistance predictions are calculated from markers reported by Pathogenwatch as described for TyphiNET [13]. For *K. pneumoniae*, resistance predictions are calculated from markers reported by Kleborate v3.2.4 [22] (run internally by Pathogenwatch [11]), according to the logic in Supplementary Table S1. For ciprofloxacin, the resistance prediction in AMRnet is taken directly from the Kleborate output, which predicts resistance to this drug based on predefined combinations of markers. For *E. coli, Shigella*, and non-typhoidal *S. enterica*, resistance predictions are calculated from markers reported by AMRfinderplus v3.11.26 [39] (run internally by Enterobase [9]), according to the logic in Supplementary Table S1. As there are not yet reliable genetic predictors of clinical resistance to specific third-generation cephalosporin, carbapenem, or aminoglycoside drugs for *Enterobacteriaceae*, we instead provide binary indicator variables for extended-spectrum beta-lactamases (ESBL), carbapenemases, and aminoglycoside resistance genes in the *K. pneumoniae, E. coli*, and *S. enterica* dashboards. The accuracy of phenotype prediction from genetic markers varies between organisms and drugs, and is an area of ongoing development and improvement. For *S*. Typhi, accuracy of resistance predictions from the genotypes used here is estimated to exceed 99.5% of all drugs, as recently reported by the Global Typhoid Genomics Consortium [40]. For *N. gonorrhoeae*, benchmarking found prediction accuracy exceeded 96% for tetracycline, benzylpenicillin, ciprofloxacin, and cefixime, but was lower for azithromycin (71.6%) and ceftriaxone (33.3%) [12]. Similar benchmarking is ongoing for the other organisms currently in AMRnet.

### Visualizations

**Figure 3.**
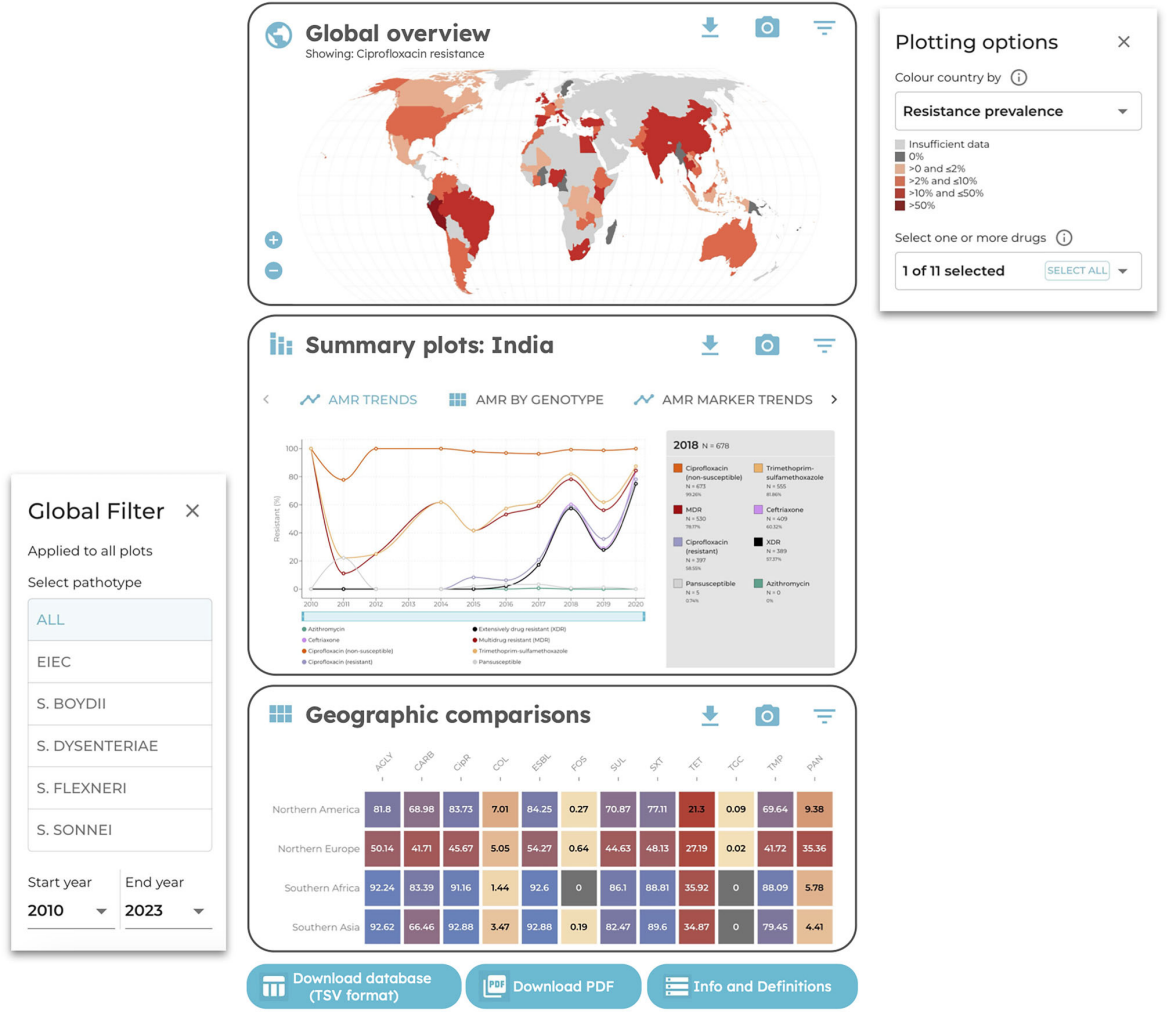
Overview of AMRnet dashboard components. Each dashboard includes three key plotting areas: global overview (map view), summary plots (designed to view details and trends for a selected country or region), and geographic comparisons (designed to compare AMR and genotype prevalence between countries or regions). Plots can be customized using global filters (which apply to all plots) and additional plot-specific options. Each plot, and its underlying summary data, can be downloaded using the camera and download icons at the top of the plotting panel. Buttons at the bottom can be used to download the full database, or a PDF report of all current plot views.

The AMRnet dashboard provides interactive visualizations to explore the prevalence of resistance mechanisms to specific drugs/classes, by country and over time, for each organism. Each dashboard has three key plotting areas (map view, summary plots, geographic comparisons), with bespoke filters and plotting options suitable to each organism (Fig. 3).

Global filters are available in a floating panel on the bottom left. Selections made here affect all plots, e.g. if a user selects a certain temporal range in the global filter, all plots will update to reflect the values for this time period only. For all dashboards, the global filters include a year range filter; for some organisms additional fields are also available for filtering (e.g. to allow users to restrict the plots to a specific diarrheagenic *E. coli* pathotype, or to carbapenemase-producing *K. pneumoniae* only; see Table 1).

The map view panel allows users to visualize AMR or genotype prevalence per country on a world map, by colouring each country according to a selected variable. The default view is to colour by resistance prevalence for the most important drug for that organism (based on the WHO Bacterial Priority Pathogens List [15]). Users can select other drugs/classes, or other variables via the ‘Plotting options’ panel. The options available are to colour by number of samples, resistance prevalence (users select which drug/s), or genotype prevalence (users select which genotype/s). Where the total number of samples passing the current global filters is below 20, prevalence values are censored and instead the country is coloured light grey to indicate insufficient data. When a user hovers over a country, a tooltip is displayed showing the total number of genomes, and the number and prevalence (percentage) of the currently selected drug/s or genotype/s, in that country. Genotype prevalences per country are plotted on a continuous scale (0%–100% prevalence). AMR prevalences are coloured on a discrete scale, to signal categorical prevalence ranges of escalating concern with respect to use of the drug for empiric therapy: (i) 0%, no resistance detected; (ii) >0% and ≤2%, resistance present but rare; (iii) >2% and ≤10%, resistance uncommon; (iv) >10% and ≤50%, resistance common; (v) >50%, established resistance.

The summary plots panel allows users to explore AMR/genotype trends and interactions in a specific region or country. A variety of plots are implemented here, with some variation depending on relevance to the organism. The plots available are: AMR annual AMR trends (line plot), annual genotype trends (stacked bar plot), AMR annual marker trends (stacked bar plot, with choice of drug to plot, and optional genotype trends overlaid), AMR frequency by genotype (heatmap), and AMR marker frequency by genotype (heatmap). For *K. pneumoniae*, there are additional plots for to explore beta-lactamase genes. For trend plots, users can select between plotting annual percentages or raw counts. Where values are plotted as percentages, values for a given year are censored if the denominator is below 10. Data are filtered according to the global filters (e.g. temporal range), and users can select a specific region or country to visualize data for; this can be selected via the ‘Plotting options’ panel or by clicking a country in the map.

The geographic comparisons panel is provided to explore how the prevalence of AMR, AMR markers, or genotypes varies between countries or regions. Data are plotted as a heatmap, with the default view being AMR prevalence per country. The ‘Plotting options’ panel can be used to stratify data (in rows) by region rather than country, to view genotype prevalence instead of AMR (in columns). For some organisms, there is also the option to plot columns as resistance markers (for a selected drug). Data are filtered according to the global filters (e.g. temporal range). Users can also use the ‘Plotting options’ to restrict the view to selected genotypes/drugs or and selected countries/regions. Countries are not plotted (and not available to select) if the number of samples passing the current global filter is below 20.

For organisms with pathotypes (i.e. *Shigella*, diarrheagenic *E. coli*, and invasive *S. enterica*), an additional pathotype comparisons panel is provided to visualize how AMR prevalence varies between pathotypes (plotted as a heatmap). The default view is AMR prevalence per pathotype, for all available pathotypes (rows) and drugs (columns). Data can be filtered to a single region or country, and users can customize the view by selecting subsets of pathotypes and drugs to include.

In each plotting area, the current view for any given plot can be downloaded as a static image file (PNG format) by clicking the camera icon, and summary values underlying the current plot (e.g. calculated annual prevalence values) can be downloaded as a tabular file (CSV format) by clicking the down arrow icon. A static report (PDF format) including the current views for all plots, and summarizing the data sources, variable definitions, and current filters, can be downloaded via a link at the bottom of the dashboard. To facilitate provenance tracking and citation of the underlying public data, dashboard users can retrieve the list of genomes, including sequence accessions and PubMed identifiers (where available), by clicking the ‘Download database’ button at the bottom of each dashboard. For *Salmonella* Typhi and *N. gonorrhoeae*, when a single country or region is selected for summary plots, the PDF reports also include a list of PubMed identifiers (or preprint DOIs) related to data from those countries.

### Unique features

The AMRnet dashboard offers numerous plot views and filters, making it highly flexible for users to explore different questions relating to the distribution of AMR and resistance determinants over time and space. Compared with the source databases (currently Pathogenwatch and Enterobase), AMRnet provides highly flexible data visualizations, focused on summarizing genome-derived AMR data by national/regional and annual prevalence. The recently released amr.watch platform [41] allows interactive exploration or AMR markers and STs from public genome data, based on the methods in Pathogenwatch; however, the platform is restricted to individual bug–drug combinations prioritized by WHO (e.g. ciprofloxacin only for *Salmonella* and *Shigella*) and lacks many features available in AMRnet.

**Figure 4.**
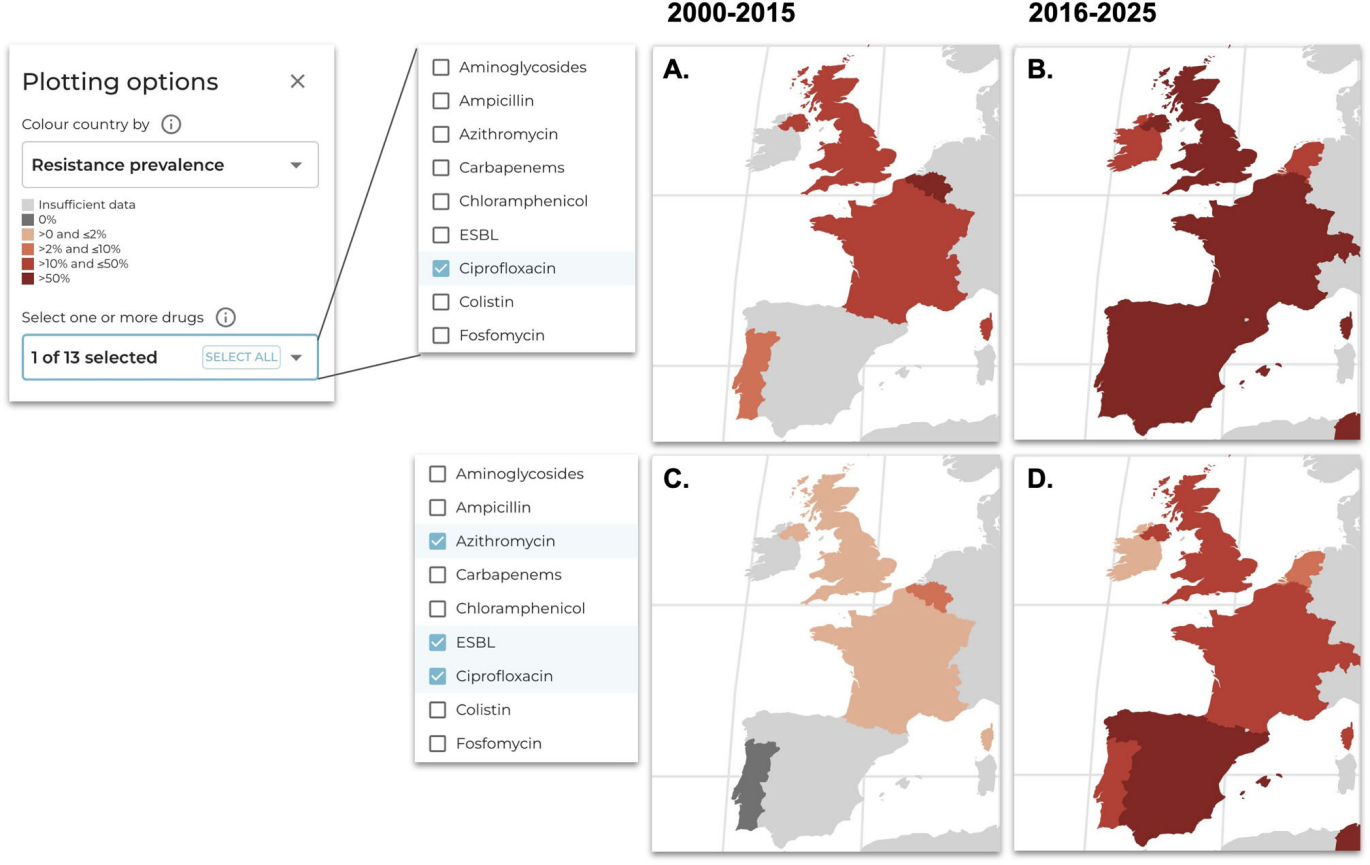
Example showing how AMRnet can be used to explore resistance to combinations of drugs. Left, plotting options for map view allow users to select one or more drugs to display resistance prevalence. Selecting a single drug (top row) results in countries being coloured by prevalence of resistance to that drug. Selecting multiple drugs (bottom row) results in countries being coloured by prevalence of resistance to the combination of all selected drugs. For example, here, by using the global filter to select *Shigella sonnei* and different time periods, we can see that ciprofloxacin resistance in *S. sonnei* has increased in Europe (**A, B**) in the last decade compared with the previous 15 years. Importantly, selecting the combination of ciprofloxacin, azithromycin, and ESBL (conferring resistance to ceftriaxone), in panel (**C**), we can see that initially ciprofloxacin-resistant strains were susceptible to the recommended drugs ceftriaxone and azithromycin, and combined resistance to all three drugs was rare in Europe (<2%) prior to 2016. Whereas in the last 10 years (**D**), this has increased such that combined resistance to all three recommended drugs is worryingly common (>20%).

A unique feature of AMRnet is the ability to examine the geographical distribution of resistance to multiple drugs in combination. This is important as, for most pathogens, combined resistance to multiple drugs is a significant problem limiting treatment options [42]. For example, ciprofloxacin is the recommended therapy for *Shigella* and *Salmonella* infections in many settings, and ciprofloxacin-resistant *Shigella* and *Salmonella* (including typhoidal and non-typhoidal, invasive or otherwise) are designated high priority by WHO [15]. However to guide responses to this problem, it is important to know what other drugs ciprofloxacin-resistant bacteria are also resistant (or susceptible) to. In the AMRnet map view, users can select one or multiple drugs to view national resistance prevalence; if multiple drugs are selected, the value plotted is the fraction of genomes that carry markers to all selected drugs. For example, for *Shigella sonnei*, the map view shows that ciprofloxacin non-susceptibility has increased in Europe (A–B) in the last 10 years compared with the previous period (Fig. 4A and B). Importantly, by viewing combinations of drugs on the map, we can see that ciprofloxacin non-susceptible strains were initially still susceptible to the second-line recommended drugs azithromycin [lacking *mph(A)* gene] and ceftriaxone (lacking ESBL genes), and combined resistance to all three drugs was rare (<2%) prior to 2016 (Fig. 4C). However in the last 10 years this has increased, and combined resistance to all three oral drugs is worryingly common in Europe (>20%) (Fig. 4D), consistent with recent reports [43].

**Figure 5.**
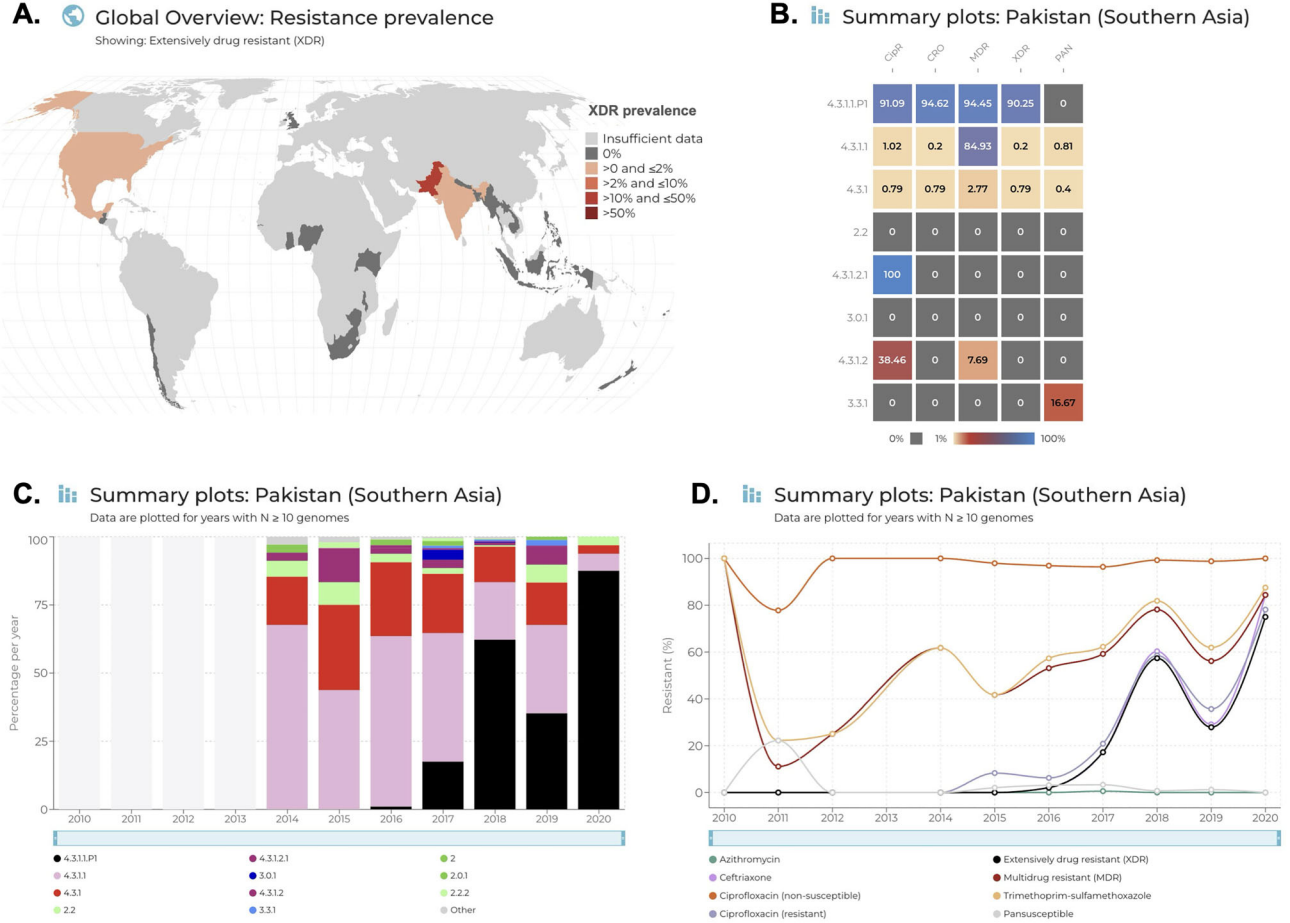
Example showing how AMRnet can be used to explore the emergence of XDR *Salmonella* Typhi in Pakistan. Global filters were set to include all isolates (travel or local) between 2010 and 2020. (**A**) Screenshot of map view, showing national XDR prevalence, which reveals Pakistan as a hotspot. (**B**) Screenshot of summary plot ‘AMR by genotype’ for Pakistan (selected by clicking on Pakistan in the map view), showing that XDR was associated almost exclusively with a single genotype, 4.3.1.1.P1. (**C**) Screenshot of ‘Genotype trends’ summary plot for Pakistan, showing that 4.3.1.1.P1 (black) was first detected in 2016 and rapidly rose in prevalence, explaining the rapid emergence of XDR since 2016, as revealed in panel (**D**), which shows a screenshot of the ‘AMR trends’ summary plot for Pakistan.

For some organisms, drug combinations of recognized concern (MDR, XDR) are precalculated and available for selection in both the map views and summary plots, making it easier to explore their emergence and spread. For example, for *Salmonella* Typhi, one can see from the map view that XDR typhoid is common in Pakistan but not elsewhere (Fig. 5A), and then dig into the summary plots to see that XDR is almost exclusively associated with a single lineage (4.3.1.1.P1) (Fig. 5B), and that this XDR lineage rapidly emerged in Pakistan in the last decade (Fig. 5C and D) [14]. Similarly, for *N. gonorrhoeae*, variables are included for MDR, XDR, and pan-susceptible to all category I/II drugs, according to the ECDC definitions [17].

**Figure 6.**
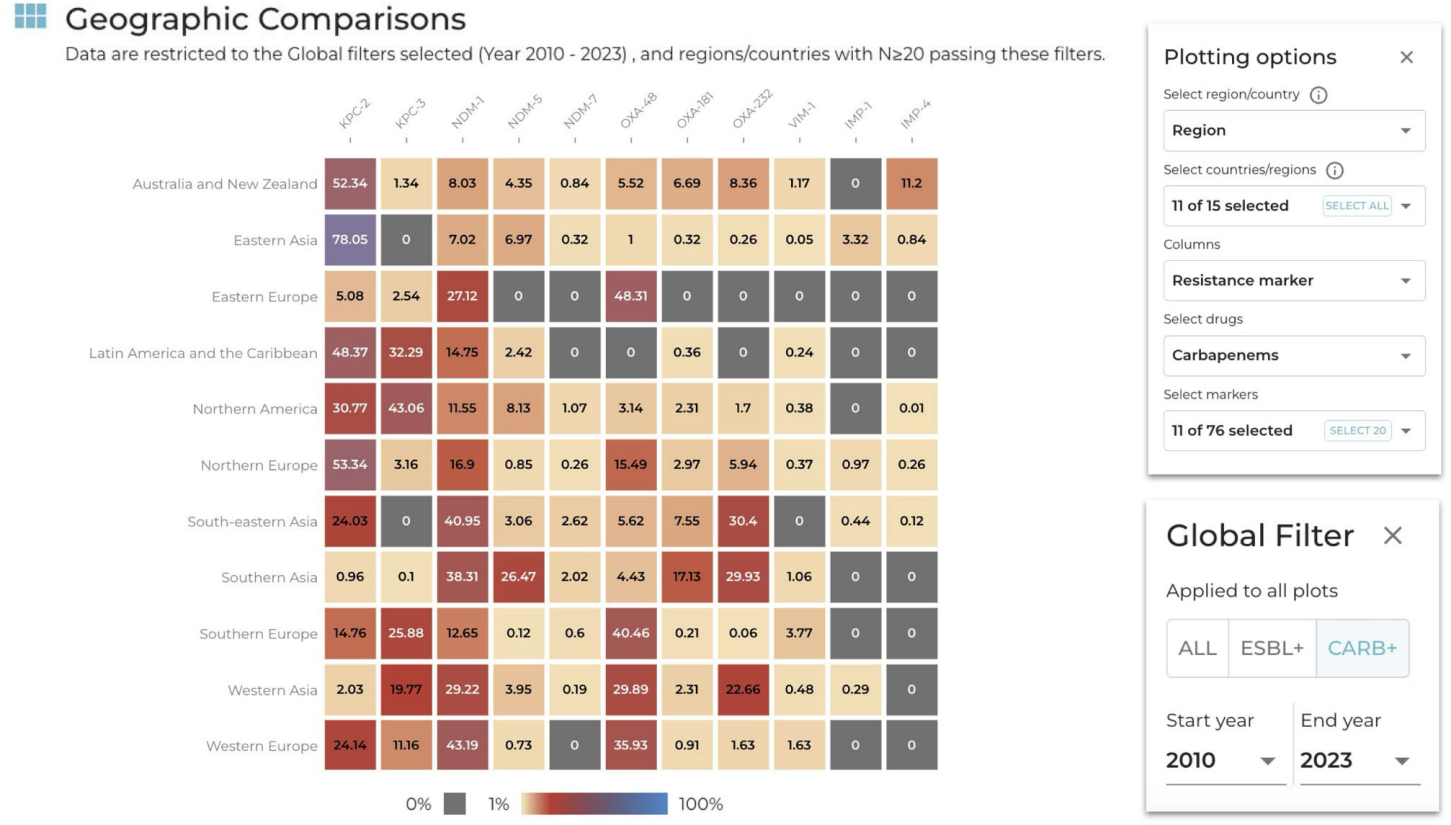
Example showing how AMRnet can be used to explore the geographical distribution of carbapenemases in *K. pneumoniae*. Screenshot of ‘geographic comparisons’ heatmap, showing prevalence of specific carbapenemase enzymes (columns) within different world regions (rows), among carbapenemase-positive *K. pneumoniae* isolated since 2010 (set using the ‘Global Filter’ as per screenshot).

Another key feature of AMRnet is the use of heatmaps to easily compare the prevalence of multiple resistances or genotypes between countries or regions. For example, by viewing ST versus region using the ‘Geographic comparisons’ panel in the the *K. pneumoniae* dashboard, with the global filter set to include only carbapenemase-positive genomes, we can see that the dominant carbapenemase-producing clones vary substantially by region; e.g. ST11 is dominant in Eastern Asia, ST258 dominates in North America, and both are common in Latin America (consistent with a recent multicentre study [44]). In the *K. pneumoniae* dashboard, it is also possible to view individual resistance markers by country or region. This makes it possible to identify regional variation in the distribution of specific carbapenemases (Fig. 6). For example, the KPC-2 and KPC-3 enzymes have different distributions: in China, Western Europe, and Northern Europe, KPC-2 is common but KPC-3 is not; whereas in Latin America, USA, and Southern Europe, both KPC-2 and KPC-3 are present at similar prevalence [44, 45]. OXA-48 is common in Eastern, Southern, and Western Europe and Western Asia, but is less common in Northern Europe and rare in other regions; in contrast, OXA-232 is common only in Asia [45, 46].

### Future developments

The utility of the AMRnet dashboard is limited by the genomic data that is available as input. The availability and representativeness of pathogen sequence data is highly variable between countries and between pathogens, depending on how widely sequencing has been adopted and how regularly data is shared publicly (if at all). The data in AMRnet should always be interpreted with this in mind, and considered alongside phenotypic data where available. To help contextualize the genome-derived information provided by AMRnet, we plan to integrate views of phenotypic data from WHO GLASS (and potentially other sources) and add metrics to indicate the number of independent sources contributing to summary statistics (in addition to the raw number of genomes as is currently presented). For example, for *S*. Typhi, where genome data was curated from different sources (national reference laboratory surveillance versus research-based surveillance in different locations), we previously reported that subsets from different sources gave very similar estimates [14].

Other planned extensions include (i) additional bacterial pathogens, including other WHO priority pathogens; (ii) additional data sources and functionality to merge data from multiple sources (e.g. enhanced metadata from a separate repository); (iii) additional functionality to explore both predetermined and user-selected combinations of resistances; and (iv) additional filters, such as source (e.g. human versus animal, blood versus urine). The latter relies on improving the quality and completeness of metadata available for public genome data, which we will aim to do in collaboration with organism expert communities (e.g. Enterobase users, the KlebNET Genomic Epidemiology Consortium), as we have done previously with the Global Typhoid Genomics Consortium [13, 14]. These efforts will also involve curating purpose-of-sampling information, so that the dashboard contents can be filtered to exclude sets of genomes not suitable for AMR surveillance (e.g. projects or programs focused on sequencing resistant strains; AMRnet currently excludes these for *S*. Typhi and *N. gonorrhoeae* only). Ingestion of other data types (e.g. phenotypic antimicrobial susceptibility data and AMR usage data from WHO GLASS; https://www.who.int/initiatives/glass), and comparisons with the genome-derived data, will also be explored. The key challenge for the future of AMRnet will be sustainability, not just in terms of digital infrastructure (code maintenance, hosting) but the availability, compatibility, and currency of surveillance-ready genome data to populate the dashboard.

Ultimately the utility of any genome-derived data for AMR surveillance will depend on continued investment in AMR and pathogen surveillance by countries and funders; ongoing commitments to sharing pathogen sequence data; and the support of ongoing global coordination efforts for AMR surveillance (e.g. via WHO GLASS), pathogen sequencing (e.g. via the WHO International Pathogen Sequencing Network), and data standards (e.g. via the Public Health Alliance for Genomic Epidemiology, PHA4GE).

## Supplementary Material

gkaf1101_Supplemental_File

## Data Availability

AMRnet is a free and open-source project. The website is accessible at https://www.amrnet.org. The underlying open-source code is available at https://github.com/amrnet/amrnet (DOI: 10.5281/zenodo.10810218). The AMRnet databases are populated from data in the public domain and can be downloaded via links at the bottom of each dashboard or programmatically via API.
