# Supplementary material for: AMRnet: a data visualization platform to interactively explore pathogen variants and antimicrobial resistance
Source: Nucleic Acids Res. 2025 Nov 6;54(D1):D691–702. doi: 10.1093/nar/gkaf1101 (PMC12807673; doi:10.1093/nar/gkaf1101)
Supplement: gkaf1101_Supplemental_File [file gkaf1101_supplemental_file.pdf]

a) Klebsiella pneumoniae			
**Resistance indicator variable**	**Definition**	**Kleborate column**	
Aminoglycosides	≥1 acquired gene	AGly_acquired	
Carbapenems	≥1 acquired carbapenemase gene	Bla_Carb_acquired	
Ciprofloxacin	Predicted NWT R	Ciprofloxacin_prediction	
Chloramphenicol	≥1 acquired gene	Phe_acquired	
Colistin	≥1 acquired gene or mutation	Col_acquired, Col_mutations	
ESBL	≥1 acquired ESBL or carbapenemase gene	Bla_ESBL_acquired, Bla_ESBL_inhR_acquired, Bla_Carb_acquired	
Fosfomycin	≥1 acquired fosA gene	Fcyn_acquired	
Macrolides	≥1 acquired gene	MLS_acquired	
Tetracycline	≥1 acquired gene	Tet_acquired	
Tigecycline	≥1 acquired gene	Tgc_acquired	
Trimethoprim	≥1 acquired dfr gene	Tmt_acquired	
Trimethoprim-Sulfamethoxazole	≥1 acquired dfr gene and ≥1 acquired sul gene	Tmt_acquired, Sul_acquired	
Pansusceptible	0 acquired resistance classes	resistance_class_count	
b) E. coli, Shigella, S. enterica			
**Resistance indicator variable**	**Definition**	**Enterobase column/s**	**Drawn from AMRfinderplus Class/Subclass**
Ampicillin	≥1 marker from any column	Penicillin, Carbapenemase, ESBL	Subclass: BETA-LACTAM
Aminoglycosides	≥1 marker	Aminoglycoside	Class: AMINOGLYCOSIDE
Azithromycin	mph(A) or acrB_R717	Macrolide	Class: MACROLIDE
Carbapenems	≥1 marker	Carbapenemase	Subclass: CARBAPENEM
Ciprofloxacin (non-susceptible)	≥1 marker	Quinolone	Class: QUINOLONE
Chloramphenicol	≥1 marker	Phenicol	Class: PHENICOL
Colistin	≥1 marker	Colistin	Class: COLISTIN
ESBL	≥1 marker from any column	ESBL, Carbapenemase	Subclass: CEPHALOSPORIN
Fosfomycin	≥1 marker	Fosfomycin	Class: FOSFOMYCIN
Tetracycline	≥1 marker	Tetracycline	Class: TETRACYCLINE
Tigecycline	≥1 marker	Other Classes: Tigecycline	Subclass: TIGECYCLINE
Trimethoprim	≥1 marker	Trimethoprim	Class: TRIMETHOPRIM
Trimethoprim-Sulfamethoxazole	≥1 marker from each column	Trimethoprim, Sulfonamide	Class: TRIMETHOPRIM, SULFONAMIDE
Pansusceptible	0 markers	all AMR columns	
